# Endobronchial Perineurioma: An Unusual Soft Tissue Lesion in an Unreported Location

**DOI:** 10.4061/2010/613824

**Published:** 2010-03-24

**Authors:** Lisa Duncan, Daryl R. Tharp, Paul Branca, Jim Lyons

**Affiliations:** ^1^Department of Pathology, University of Tennessee Medical Center, 1924 Alcoa Highway, Knoxville, TN 37920, USA; ^2^Department of Medicine, University of Tennessee Medical Center, 1924 Alcoa Highway, Knoxville, TN 37920, USA; ^3^AmeriPath, Indianapolis, IN 46219, USA

## Abstract

We report the first case of an endobronchial perineurioma, a rare benign neoplasm typically occurring in soft tissue. A 53-year-old nonsmoking female presented with a three-month history of persistent bronchitis. A CT scan followed by bronchoscopy demonstrated an endobronchial lesion involving the left mainstem bronchus. Removal of the lesion by bronchoscopy was accomplished. The tumor was composed of bland spindle cells in a variably collagenized stroma. These cells had long cytoplasmic processes. No mitotic activity or necrosis was observed. Neoplastic cells were immunoreactive for epithelial membrane antigen (EMA), CD34, and claudin-1. Smooth muscle actin (SMA), desmin, and S-100 immunostains were all negative. Based on the morphologic appearance and immunophenotype, a diagnosis of perineurioma was rendered.

## 1. Introduction

Perineuriomas are rare benign neoplasms representing a proliferation of perineurial cells. Two distinct subtypes are recognized and include soft tissue perineurioma and intraneural perineurioma. Perineuriomas most commonly occur in the dermis and subcutis of the limbs or trunk, but other locations have been reported. Herein, we describe the first case of soft tissue perineurioma occurring in an endobronchial location.

## 2. Case Report

**Figure 1:**
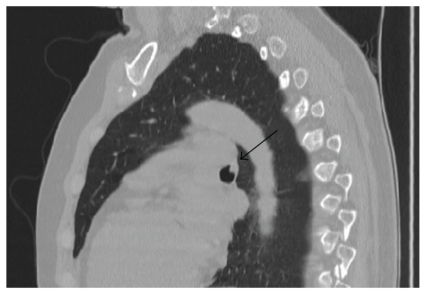
Three dimensional reconstructed CT image demonstrating an endobronchial soft tissue abnormality.

A 53-year-old nonsmoking female presented to her primary care physician with a three-month history of a nonresolving upper respiratory infection. A chest-computed tomographic (CT) scan demonstrated a 6 mm endobronchial soft tissue abnormality with slight contrast enhancement at the periphery involving the left mainstem bronchus. Three-dimensional reconstruction of CT images showed similar findings (Figure 1). 

**Figure 2:**
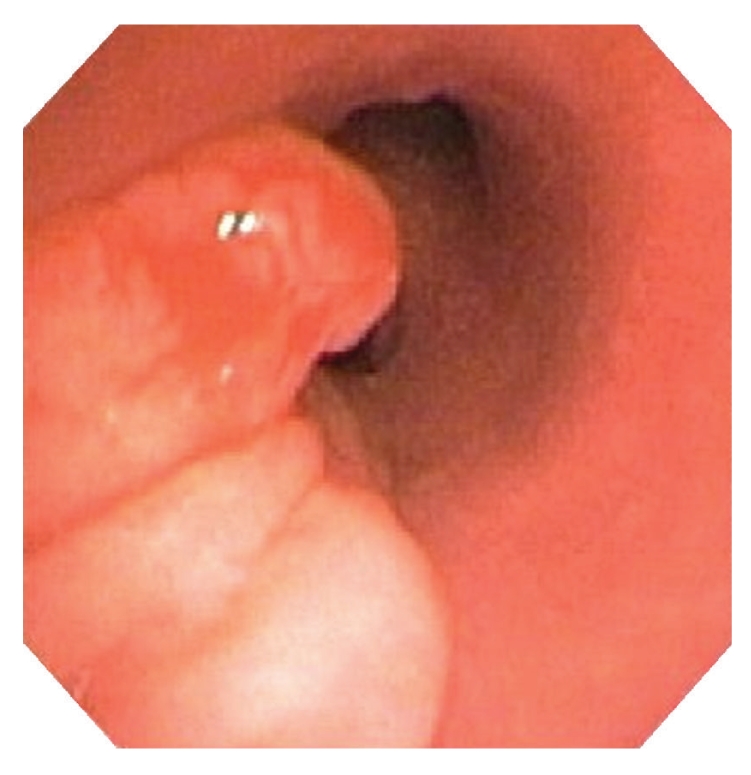
Bronchoscopic image demonstrating an endobronchial nodule at the left mainstem bronchus.

Bronchoscopic evaluation showed an endobronchial nodule involving the left mainstem bronchus (Figure 2). The lesion was removed during a subsequent bronchoscopy. A CT scan, including three dimensional bronchial reconstruction performed after a six month interval, showed no evidence of a residual endobronchial lesion. The patient is well one year following initial presentation.

## 3. Materials and Methods

Bronchoscopic biopsies were fixed in 10% neutral buffered formalin then subjected to routine processing and paraffin embedding. Sections were stained with hematoxylin and eosin. Immunohistochemical stains were performed on paraffin embedded tissue using the avidin biotin peroxidase complex method (DakoCytomation Autostainer, Denmark). Antibodies used are shown in Table 1.

## 4. Pathologic Findings

**Figure 3:**
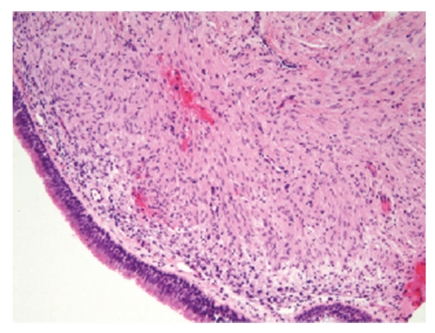
Spindle cell proliferation situated beneath benign bronchial mucosa. Hematoxylin and eosin.

**Figure 4:**
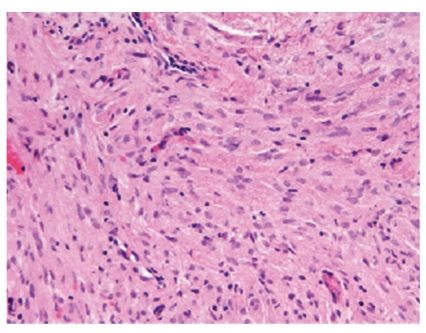
Photomicrograph demonstrating a bland spindle cell proliferation with a collagenized stroma. Hematoxylin and eosin.

**Figure 5:**
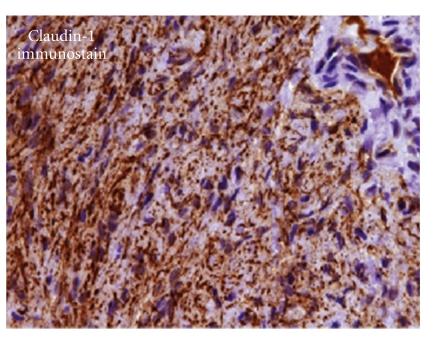
Spindle cells staining positively with claudin-1.

**Figure 6:**
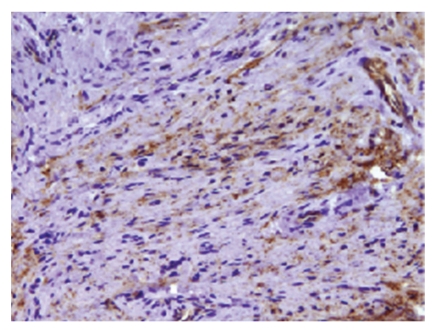
Spindle cells staining positively with epithelial membrane antigen (EMA).

Sections of bronchial biopsies demonstrated a cellular proliferation that was situated in submucosal tissue beneath histologically unremarkable bronchial mucosa (Figure 3). The unencapsulated proliferation of cytologically bland elongated spindle cells was arranged in a storiform pattern within a collagenized stroma (Figure 4). No nuclear pleomorphism, mitotic activity, or necrosis were noted. The spindle cell population expressed positivity for CD34, claudin-1 (Figure 5), and epithelial membrane antigen (EMA) (Figure 6). The spindle cell population showed no staining for cytokeratin (AE1/AE3), desmin, smooth muscle actin (SMA), S-100, or CD117.

## 5. Discussion

Perineurioma is a rare benign tumor composed exclusively of perineurial cells. First reported by Lazarus et al., it is typically characterized by an unencapsulated yet circumscribed proliferation of bland spindle cells arranged in a storiform pattern within a variably collagenized stroma. Ultrastructural analysis demonstrates thin bipolar cytoplasmic processes, junctional complexes, and smooth vesicles [1, 2]. In the largest case series reported, Hornick and Fletcher reviewed 81 cases of perineurioma. The majority of their cases occurred in the dermis and subcutis of the limbs and trunk, having a mean age of presentation in middle age with a slight female predominance [1]. Hornick and Fletcher separately reported ten cases in the intestinal tract (nine colonic and one jejunal) which presented as either polyps or submucosal mass lesions [3]. Perineuriomas have furthermore been reported in the stomach [4], kidney [5–7], lip [8], maxillary sinus [8] and mandible [9]. Giannini et al. reported an intraventricular perineurioma [10]. Perineurioma occurring in an endobronchial location has never been reported. 

Soft tissue and intraneural subtypes of perineurioma have been described. Soft tissue perineurioma itself has three types. The sclerosing variant of soft tissue perineurioma presents as a small painless dermal or subcutaneous mass involving the digits or palms of young adults [11]. It is unique due to the presence of epithelioid perineurial cells, extensive collagenization of the stroma and trabecular growth pattern. It differs from fibroma of tendon sheath which is associated with tendons. Sclerotic fibroma expresses factor XIIIa and collagen IV but not epithelial membrane antigen (EMA) as would be seen in sclerosing perineurioma. The reticular variant of soft tissue perineurioma encompasses a group of perineuriomas having a prominent lace-like reticular arrangement of lesional cells within a variably myxoid stroma [12]. The differential diagnosis of these lesions includes myoepithelial tumors, ossifying fibromyxoid tumor, extraskeletal myxoid chondrosarcoma, myxoid malignant peripheral nerve sheath tumor, and myxoid synovial sarcoma. The presence of EMA positivity helps to differentiate the reticular variant of soft tissue perineurioma from these other entities in most cases. While myxoid synovial sarcoma is EMA positive, it also tends to be positive for CD99 and bcl-2, markers which would be absent in perineurioma. Finally, plexiform soft tissue perineurioma is a very rare variant which must be distinguished from multiple neoplasms having a plexiform pattern of growth such as plexiform neurofibroma, plexiform schwannoma, and plexiform circumscribed neuroma [13]. All of these tumors are typically S-100 positive and EMA negative in contrast to perineurioma. In general, soft tissue perineurioma must also be differentiated from dermatofibrosarcoma protuberans (DFSPs), low-grade fibromyxoid sarcoma (LGFMS), desmoid tumor, fibromatosis, and nerve sheath tumors [14]. Biphasic tumors having a mixture of either schwannoma or neurofibroma, and perineurioma have also been described and can present particular difficulty [15]. There are also striking similarities between soft tissue perineurioma and cutaneous meningioma, which in some instances, can only be resolved by electron microscopy [16]. Since cases of soft tissue perineurioma have been reported in the intestinal tract, gastrointestinal stromal tumor should be excluded with a CD117 stain.

The second subtype of perineurioma is the intraneural perineurioma, formerly referred to as localized hypertrophic neuropathy [14, 17]. It has a distinct clinical presentation, usually limited to a segment of nerve in the extremities. It is characterized by complex wrapping of neoplastic perineurial cells around endoneurial structures, resulting in the formation of pseudo-onion bulbs. Involvement of multiple nerve fascicles produces a rope-like thickening of the nerve which often results in loss of sensorimotor function. Malignant transformation has not been reported, and it is not associated with neurofibromatosis. 

Immunohistochemistry is pivotal in the accurate classification of perineurioma. The majority of perineuriomas express EMA with variable expression of CD34 and smooth muscle actin (SMA). Glut-1 is a human red blood cell glucose transporter which is expressed in perineurial cells. In their study, Yamaguchi et al. demonstrated strong reactivity for glut-1 in the five cases they reported [18]. No staining for glut-1 was found for schwannoma, neurofibroma or cutaneous meningioma. The presence of claudin-1 staining in perineuriomas has been reported to be 29% [10] to 92% [19]. Claudins are a family of proteins that function in the structure of the tight junction, and claudin-1 is expressed in many body site locations. Folpe et al. did not find claudin-1 reactivity in DFSP, LGFMS, or fibromatosis; therefore, they concluded that claudin-1 could be utilized in distinguishing perineurioma from its mimics [19]. 

The clonal nature of intraneural perineurioma has been confirmed by demonstrating a lack of all or a portion of chromosome 22 [17]. Deletion in chromosome 22 in soft tissue perineurioma has also been reported and suggests that soft tissue and intraneural perineuriomas represent a spectrum of perineurial neoplasia [14]. Deletion of chromosome 13 has also been described in a large soft tissue perineurioma [9]. 

Perineurioma is thought to represent a benign entity. Hirose et al. reviewed 121 malignant peripheral nerve sheath tumors (MPNSTs) from their files looking for cases having perineurial differentiation [20]. A total of 23 cases were selected for study based on the histologic findings of spindle cells having long cytoplasmic processes present in whorls or storiform arrangements typical of that seen in perineurioma. These cases were subjected to immunostaining, and five cases were found to have tumor cells which were S-100 negative and EMA positive. These authors emphasized the importance of delineating perineurial differentiation in MPNST because these tumors tend to have a more favorable prognosis than conventional MPNST. 

The case presented in this paper represents an endobronchial perineurioma. While perineurioma usually occurs in the skin and superficial soft tissues, it has been reported in unusual sites and should be considered in the differential diagnosis of spindle cell lesions in any location.

## Figures and Tables

**Table 1:** Panel of antibodies used for immunohistochemical analysis.

Antibody	Clone	Company	Antigen retrieval	Dilution
Cytokeratin	AE1/AE3	Dako	Steam	Prediluted
Desmin	DE-R-11	Dako	Steam	Prediluted
EMA	Monoclonal	Dako	Citrate buffer	Prediluted
CD34	QBEnd 10	Dako	Steam	1 : 25
SMA	1A4	Dako	None	Prediluted
S100	Polyclonal	Dako	Steam	Prediluted
MyoD1	5.8A	Dako	Steam	1 : 50
CD117	T595	Biogenex	Steam	Prediluted
Claudin-1	Polyclonal	Zymed	Steam	Prediluted
